# Correlation between Flow Temperature and Average Molar Ionic Potential of Ash during Gasification of Coal and Phosphorus-Rich Biomass

**DOI:** 10.3390/molecules28237858

**Published:** 2023-11-30

**Authors:** Chaoyue Zhao, Qingyun Wang, Xiaoyong Men, Yuchen Li, Linmin Zhang, Yonghui Bai, Xudong Song, Jiaofei Wang, Min Yao, Guangsuo Yu

**Affiliations:** 1 State Key Laboratory of High-Efficiency Utilization of Coal and Green Chemical Engineering, College of Chemistry and Chemical Engineering, Ningxia University, Yinchuan 750021, China; zhaochaoyue2019@163.com (C.Z.); wangqingyun0905@163.com (Q.W.); soyyyaha@163.com (Y.L.); linminzhangnx@163.com (L.Z.); xdsong@nxu.edu.cn (X.S.); wjfdafei@nxu.edu.cn (J.W.); 2 CHN Energy Ningxia Coal Industry Co., Ltd., Yinchuan 750000, China; 15011901@chnenergy.com.cn; 3 Institute of Clean Coal Technology, East China University of Science and Technology, Shanghai 200237, China; gsyu@nxu.edu.cn

**Keywords:** average molar ionic potential, biomass, CaO/P_2_O_5_, coal, co-gasification, flow temperature, mineral, prediction

## Abstract

The co-gasification of biomass and coal is helpful for achieving the clean and efficient utilization of phosphorus-rich biomass. A large number of alkali and alkaline earth metals (AAEMs) present in the ash system of coal (or biomass) cause varying degrees of ash, slagging, and corrosion problems in the entrained flow gasifier. Meanwhile, phosphorus is present in the slag in the form of ${\mathrm{PO}}_{4}^{3^{-}}$, which has a strong affinity for AAEMs (especially for Ca^2+^) to produce minerals dominated by calcium phosphates or alkaline Ca-phosphate, effectively mitigating the aforementioned problems. To investigate the changing behavior of the slag flow temperature (FT) under different CaO/P_2_O_5_ ratios, 72 synthetic ashes with varying CaO/P_2_O_5_ ratios at different Si/Al contents and compositions were prepared, and their ash fusion temperatures were tested. The effects of different CaO/P_2_O_5_ ratios on the FT were analyzed using FactSage thermodynamic simulation. A model for predicting slag FT at different CaO/P_2_O_5_ ratios was constructed on the basis of the average molar ionic potential (I_a_) method and used to predict data reported from 19 mixed ashes in the literature. The results showed that I_a_ and FT gradually increased with a decreasing CaO/P_2_O_5_ ratio, and the main mineral types shifted from anorthite → mullite → berlinite, which reasonably explained the decrease in ash fusion temperatures in the mixed ash. The established model showed good adaptability to the prediction of 19 actual coal ash FTs in the literature; the deviation of the prediction was in the range of 40 °C. The model proposed between FT and I_a_ based on the different CaO/P_2_O_5_ ratios can be used to predict the low-rank coal and phosphorus-rich biomass and their mixed ashes.

## 1. Introduction

The entrained flow bed (EFB) gasification technology has various advantages, including diversified feedstock (coal, biomass, petroleum coke, solid waste, etc.), high conversion efficiency, a wide operating temperature range, and low pollutant emissions. Therefore, it is a highly promising gasification technology for coal/biomass gasification [1,2]. Waste carbonaceous biomass, such as sewage sludge and animal manure, contains a high amount of alkali and alkaline earth metals (AAEMs, including K, Na, Ca, Mg, etc.), as well as phosphorus elements. Issues such as ash build-up, caking, sintering, and slagging can occur in the gasifier when gasification is carried out separately [3,4]. Co-gasification of this phosphorus-rich biomass (with a P_2_O_5_ content exceeding 10% by mass) with coal not only offers a clean, efficient, and effective way to utilize the phosphorus-rich biomass but also helps regulate the slag flow behavior of the coal during gasification. This regulation is achieved due to the high content of CaO and P_2_O_5_ in the ash [5].

To date, numerous scholars have studied the mechanism of mineral transformation involving phosphorus and other elements during the thermal conversion of phosphorus-rich biomass, such as corn stover [6], jatropha seed cake [7], municipal sewage sludge [8], and livestock manure (including cattle and swine manure) [9], along with phosphorus-based additives [3,10,11,12,13] in combination with coal or biomass of different ash compositions. The fusion behavior of ash/slag is dependent on the chemical composition of the coal ash and the thermal transformation of minerals at high temperatures [14]. When investigating the mechanism of the slag fluidity for coal and biomass, it is preferable to employ a single phosphorus-based additive. For example, Yu et al. [10] discovered that ${\mathrm{PO}}_{4}^{3^{-}}$ found in struvite (magnesium ammonium phosphate, MgNH_4_PO_4_) effectively captured sodium and calcium in Zhundong coal. This capturing action inhibited sodium and calcium from forming low-temperature eutectic mixtures with silica and aluminum, which improved ash accumulation during the combustion process of Zhundong coal. It is known that the ionic potential of P^5+^ (147 nm^−1^) is higher than that of Si^4+^ (92 nm^−1^), and as a result, P^5+^ preferentially combines with Al^3+^ in the form of ${\mathrm{PO}}_{4}^{3^{-}}$ to form berlinite tetrahedra within the system. This inhibits the formation of silica–aluminum salts, such as mullite and anorthite. At the same time, phosphorus has a high affinity for AAEMs, especially in the slag system of calcium minerals (anorthite, hedenbergite, grossularite, etc.), which captures Ca^2+^ to form calcium phosphate salts [5]. Li et al. [11] conducted a study using P_2_O_5_ to modulate the ash fusion characteristics of three different coals with varying ash fractions: Shazhuzi coal with a high silica–aluminum content, Zhundong coal with a high calcium content, and Yimin coal with a high calcium–iron content. The addition of P_2_O_5_ to Shazhuzi coal ash resulted in an increase in berlinite content, the inhibition of mullite formation, and a decrease in ash fusion temperatures (AFTs); in Zhundong and Yimin coals, the presence of P_2_O_5_ led to the formation of calcium phosphates, resulting in higher AFTs. In addition, Li et al. [2] used phosphogypsum (Ca_5_F(PO_4_)_3_) to investigate the mechanism of Xiaolongtan coal (a high-calcium, high-sulfur coal) AFTs. The study revealed that as the phosphogypsum content increased, anorthite transformed into high-melting-point minerals, such as Ca_2_Al_2_SiO_7_, Ca_3_SiO_5_, Ca_5_P_2_SiO_12_, and Ca_7_MgSi_4_O_16_, increasing the AFTs.

Similarly, phosphorus-based additives can be utilized to mitigate the release of K and Na from biomass, inhibit shrinkage and melting, and enhance the AFTs of the biomass. The presence of phosphorus in the slag system can be represented by the Al_2_O_3_/(CaO + Na_2_O + K_2_O) (A/CNK) molar ratio. It is a peralkaline system when A/CNK < 1 and phosphorus combines with alkaline elements to form a phosphate structure, thus increasing polymerization. In a weak alkaline system (A/CNK ≈ 1), the A/CNK ratio remains approximately equal to 1. An excessive aluminum system remains when A/CNK > 1, berlinite is formed, and the chain length of the Si-O skeleton structure decreases, decreasing the AFTs and the viscosity. To investigate the phosphorus capture or inhibition of specific alkali metal morphological changes, the molar ratio of the mixed ash components is usually varied to explain the mechanism [3,8]. Zhu et al. [6] studied the impact of two additives, Ca(H_2_PO_4_) and NH_4_H_2_PO_4_, on the sintering properties of corn stover ash slag at different P/K molar ratios (0.5, 1, and 2). They discovered that as the P/K molar ratio increased, a significant number of potassium-rich silicate ash aggregates gradually transformed into high-melting-point calcium–phosphate salts or potassium–calcium–phosphate salts. Additionally, Zhang et al. [5] found that phosphorus also bound Ca^2+^ to form sodium–calcium–phosphate when capturing Na^+^.

On the basis of the information above, it can be observed that phosphorus in different types of coal ash generates calcium phosphate or alkaline calcium–phosphate salts with Ca^2+^, indicating a strong affinity between phosphorus and Ca^2+^. However, few studies have investigated the fusion characteristics of phosphorus-containing ash/slag at a reducing atmosphere and explored slag flow temperatures (FT) using different CaO/P_2_O_5_ ratios (mass ratio). The lack of work could significantly restrict the gasification of this fuel [6]. FT is an important parameter for the EFB gasifier to operate steadily. Traditionally, the FT value is determined by creating a standard ash cone from coal ash and observing its melting degree during the heating process using an ash melting point tester. Nevertheless, due to the harsh conditions at high temperatures, high pressure, and turbulent multi-phase reactions inside the EFB gasifier, it is challenging to obtain accurate slag flow behavior parameters, which makes it difficult to determine the FT [15]. Since the slag melting process is dynamic, a single stable parameter must be related to FT. The average molar ion potential (I_a_) reflects the polymerization of the slag structure and assesses the stability of coal ash on the basis of the variation of oxygen atom species and quantity [16]. Therefore, the use of I_a_ as a stable variable parameter holds some general applicability in predicting the melting behavior of coal ash [17]. A distinct linear relationship between FT and I_a_ has been observed, enhancing the accuracy of FT prediction [18]. As a result, the FT of CaO/P_2_O_5_ slags with different ratios can be predicted by constructing an FT-I_a_ model. Coal and biomass, with varying ash compositions, exhibit different fusion behaviors. Mechanisms are different in regulating their mixed ash at different CaO/P_2_O_5_ ratios. Typically, coal/biomass ashes are classified into three types: low-silica–alumina (SiO_2_ + Al_2_O_3_ ≤ 65%, referred to as S + A, mass ratio), medium-silica–alumina (S + A = 65–75%), and high-silica–alumina ash (S + A ≥ 80%).

In this work, 72 synthetic ashes with varying Si/Al contents and ratios and different CaO/P_2_O_5_ ratios (10:0, 8:2, 6:4, 4:6, 2:8, and 0:10) were prepared and tested to determine their FTs. Furthermore, FactSage 7.3 thermodynamic simulation software was utilized to investigate the mechanism through which different CaO/P_2_O_5_ ratios affected the FT of these 72 synthetic ashes. A model was then constructed to predict the FT of different ash types under various CaO/P_2_O_5_ ratios. This is instructive for the prediction of the FT of mixed ash during the co-gasification of coal and phosphorus-rich biomass.

## 2. Results and Discussion

### 2.1. Effect of CaO/P_2_O_5_ Ratios on the I_a_ of Synthetic Ashes

**Figure 1:**
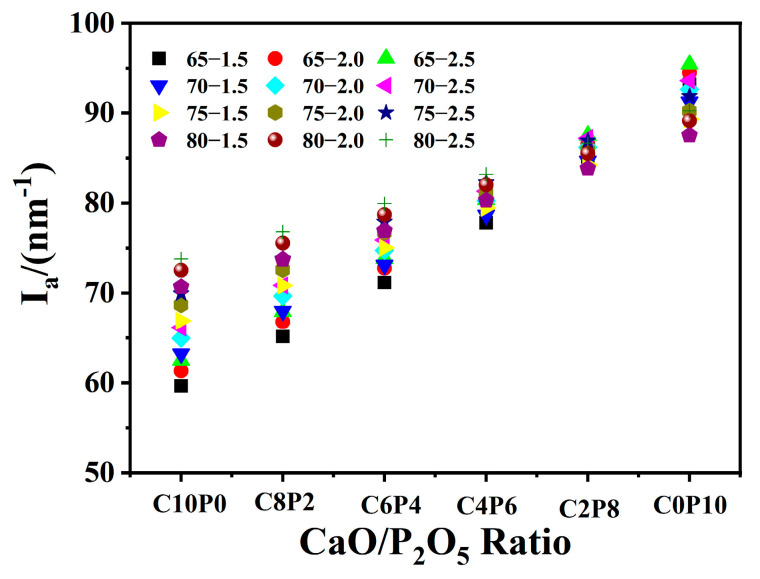
I_a_ variation of 72 synthetic ashes with different CaO/P_2_O_5_ mass ratios.

Figure 1 illustrates the influence of different CaO/P_2_O_5_ ratios on the I_a_ trend for 72 synthetic ashes. With an increasing P_2_O_5_ content in the CaO/P_2_O_5_ ratio, I_a_ showed an upward trend under the same S + A and S/A ratios, and the I_a_ value gradually increased. Different values of I_a_ were obtained for the same CaO/P_2_O_5_ ratio when the total S + A and S/A ratios were different in synthetic ash. Compared with Figure 1, the I_a_ values for different S/A ratios showed a trend of I_(S/A=1.5)_ > I_(S/A=2.0)_ > I_(S/A=2.5)_ at the same total S + A. At the same S/A ratio, the I_a_ values at the C10P0 and C8P2 ratios increased as the total amount of S + A summation rose, and the I_a_ values at the C6P4, C4P6, C2P8, and C0P10 ratios decreased as the total amount of S + A summation rose. The reason for this phenomenon is that the proportion of CaO and P_2_O_5_ was relatively reduced when the total amount of S + A increased from 65 to 80. It is known that the metal ionic potentials of the five oxides are I_P_^5+^ > I_Si_^4+^ > I_Al_^3+^ > I_Fe_^2+^ > I_Ca_^2+^ [19], which leads to a gradual decrease in the I_a_ value.

### 2.2. Effects of CaO/P_2_O_5_ Ratios on the FT of Synthetic Ash

#### 2.2.1. Variation of FT

**Figure 2:**
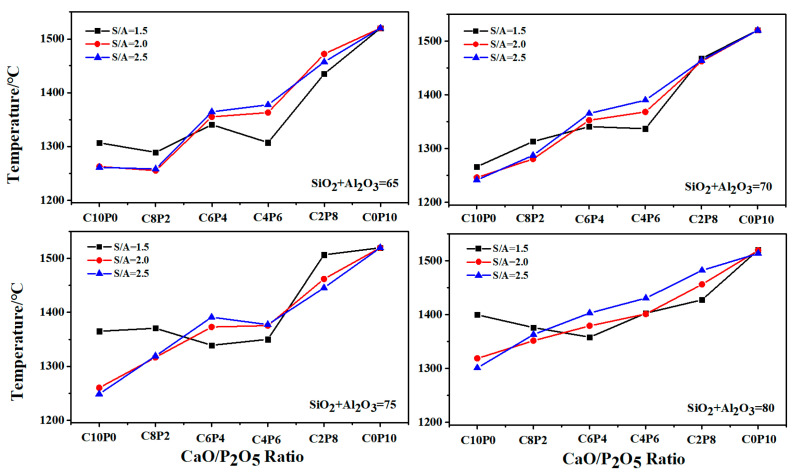
AFT changes of 72 synthetic ashes with different CaO/P_2_O_5_ mass ratios.

The variation trends of the FT for 72 synthetic ashes with the increase in P_2_O_5_ content in the CaO/P_2_O_5_ ratio are shown in Figure 2. It can be observed that the variation curves for four different total amounts of S + A (65, 70, 75, and 80) displayed an upward trend. The range of FT temperature variations for the three different S/A ratios at each ratio was similar. Specifically, the variation trends of FT for synthetic ashes with S/A ratios of 2.0 and 2.5 were identical, both exhibiting a stepwise upward trend. The variation curve of FT can be divided into three segments: (1) in the C10P0, C8P2, and C6P4 stages, there was a gradual and slow rising trend, with FT values below 1350 °C; (2) the C6P4 and C4P6 stages tended to flatten out, with FT values ranging between 1350 °C and 1400 °C; and (3) in the C4P6, C2P8, and C0P10 stages, there was a linear increase, with FT values exceeding 1400 °C. The mineral phase interactions caused by the interaction between the constituents of the slag at high temperatures were the main reason for the different variations in FT changes.

Since the compositions and contents of coal ash were different, the minerals produced were different, and the slags had different reticulation and stability, resulting in different I_a_ and FT values. The higher the I_a_ value, the greater the stability of the slag, which means a higher FT.

#### 2.2.2. Mechanism of the Effect of CaO/P_2_O_5_ Ratios on the FT of Synthetic Ash

FactSage 7.3 thermodynamic simulation software follows the principle of Gibbs energy minimization to determine whether the formation of a solid phase is energetically favorable [20]. To explore the mechanism of the influence of different CaO/P_2_O_5_ ratios on the FT of synthetic ashes from the thermodynamic aspects of the state and properties of ash slag at high temperatures, the changes in the multivariate phase equilibrium and mineral melting process of 72 synthetic ashes at different temperatures were calculated by the simulation.

**Figure 3:**
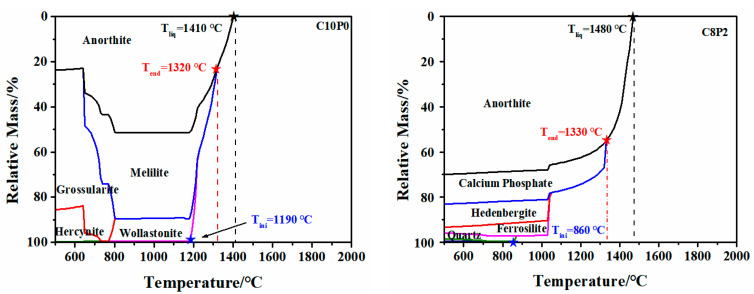

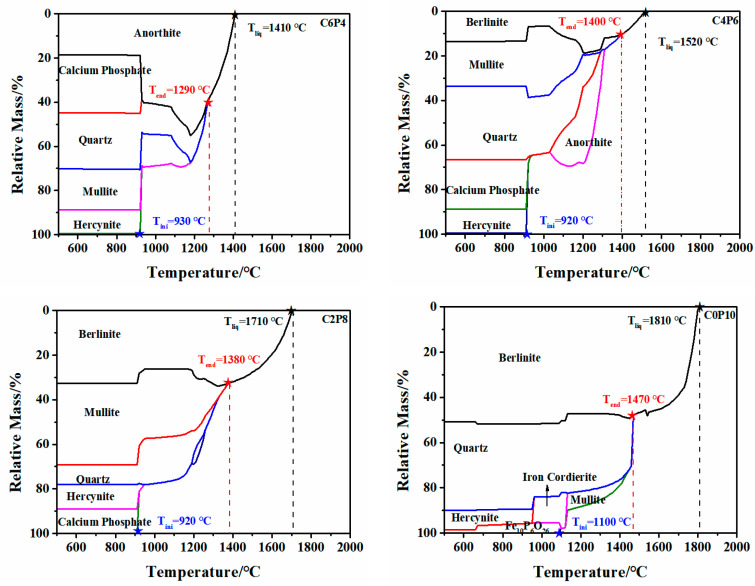
The analysis of phase assemblage–temperature curves for six different CaO/P_2_O_5_ synthesized ashes under 65-1.5 conditions. Black star and line is T_liq_; red star and line is T_end_; blue star and line is T_ini_. Quartz–SiO_2_; Mullite–Al_6_Si_2_O_13_; Anorthite–CaAl_2_Si_2_O_8_; Hercynite–FeAl_2_O_4_; Grossularite–Ca_2_Al_2_SiO_7_; Melilite–Ca_2_FeSi_2_O_7_; Hedenbergite–CaFe(SiO_3_)_2_; Calcium phosphate–Ca_5_P_3_HO_13_; Ferrosilite–Fe_2_Si_2_O_6_; Berlinite–AlPO_4_.

Take the compositions, contents, and corresponding parameters (initial liquid phase temperature (T_ini_), full liquid phase temperature (T_liq_), temperature of the last mineral in the solid phase (T_end_)) and the variations of six different CaO/P_2_O_5_ synthetic ash minerals under S + A = 65 and S/A = 1.5 as an example. As shown in Figure 3, different minerals were generated at high temperatures due to the different CaO and P_2_O_5_ contents at the different CaO/P_2_O_5_ ratios, which could be roughly divided into skeletal silica-aluminum minerals (mullite and quartz), calcium minerals (anorthite, grossularite, melilite, hedenbergite, and wollastonite), phosphate minerals (calcium phosphate, and berlinite) [3,21]. Large amounts of Ca^2+^ lead to the depolymerization of the SiO_4_ tetrahedra, while P^5+^ increases the stability of the polymer structure in the slag [22,23]. Minerals produced under the C10P0 ratio were calcium minerals; anorthite (1550 °C) is categorized as a calcium mineral with a higher melting point and is often used as a sub-liquid phase to determine T_end_. Among these, the anorthite content determines the value of T_liq_, resulting in a lower FT in the calcium system. As the total amount of S + A increased, the mineral composition gradually changed from calcium minerals to skeletal minerals and quartz crystals and then separated, and the proportion of calcium minerals decreased, resulting in a gradual increase in T_end_ and T_liq_, which is why the FT exhibited a smoother or slower growth state under the C6P4 and C4P6 ratios. With an increase in P_2_O_5_ content, phosphorus exists in the system in the form of ${\mathrm{PO}}_{4}^{3^{-}}$ and tends to bind with AAEMs [3]. As a result, calcium phosphate occurs in the proportions of C8P2, C6P4, C4P6, and C2P8. It is known that I_P_^5+^ > I_Si_^4+^, which also preferentially binds free Al^3+^ in the system to form berlinite, so T_liq_ reaches around 1800 °C.

The variation of AFTs is influenced by mineral reactions and liquid phase diffusion in coal/biomass ash [24]. Additionally, AFTs can be predicted on the basis of thermodynamic parameters. Tini represents the maximum temperature at which the initial liquid phase appears in the system, while T_liq_ represents the minimum temperature at which the last solid phase disappears (Figure 3). The difference value between these two temperatures defines the entire interval of the melting temperature range in which all minerals in the slag completely convert to the liquid phase [2]. Two parameters, ΔT_1_ (predicted temperature deviation: ΔT_1_ = T_liq_ − T_ini_, where the values of the parameters T_liq_ and Tini were obtained from Figure 3) and ΔT_2_ (actual temperature deviation: ΔT_2_ = FT − DT), demonstrate the range of variation in the slag melting process. The magnitude of these temperature differences can be used to explain the mechanism of variation in terms of the increase or decrease in FT [25]. 

**Figure 4:**
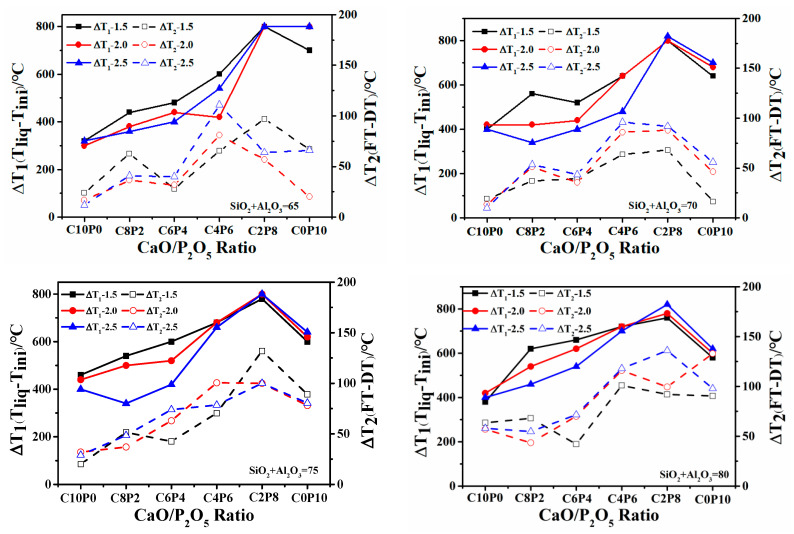
The analysis of ash melting range for 72 synthetic ashes at different temperatures.

Figure 4 shows the graphs of the ash melting range temperature differences with CaO/P_2_O_5_ ratios for the four S + A synthetic ashes, and the comparison shows that both ΔT_1_ and ΔT_2_ showed different increasing trends with an increasing P_2_O_5_ content in CaO/P_2_O_5_, and the melting range was broadened, which could also explain the increased FT. It is known that the FT is lower than 1400 °C, ΔT_1_ is 400–600 °C, and ΔT_2_ < 75 °C in the intervals of C10P0, C8P2, and C6P4; ΔT_1_ and ΔT_2_ drop and intersect to converge at the same point at C6P4 to reach the lowest melting temperature at S + A = 65 and 70. At C4P6, C2P8, and C0P10, ΔT_1_ > 600 °C and ΔT_2_ > 75 °C. At C2P8 and C0P10, the curves tended to decrease, which was attributed to the fact that the upper limit of the use temperature of the AFT analyzer was 1520 °C, and thus the FT of some high-melting-point synthetic ashes could not be accurately measured. Even though ΔT_2_ retains some error at FT > 1520 °C, the prediction mechanism of phosphorus-rich biomass co-gasification with coal can still be explained by two parameters (ΔT_1_ and ΔT_2_).

**Figure 5:**
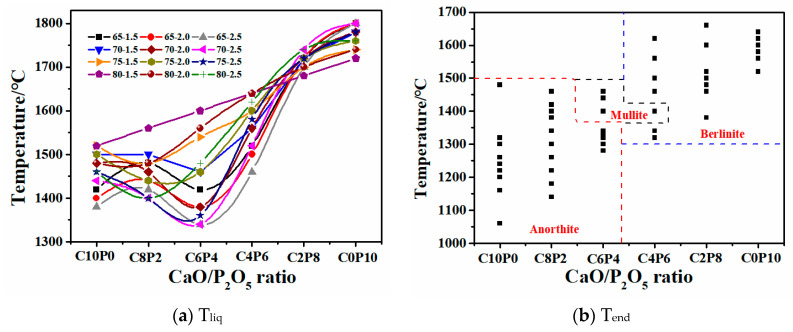
The variation of T_liq_ and Tend for 72 synthetic ashes. Dotted lines—areas of anorthite, berlinite, and liquid phase minerals; black square—72 synthetic ashes.

To further investigate the mechanism of the effect of different CaO/P_2_O_5_ ratios on AFTs, the variation of the liquid phase content with different CaO/P_2_O_5_ ratios (Figure 5a) and the last plot of the variation of the solid phase partitioning of the minerals (Figure 5b) were comparatively analyzed. As shown in Figure 5a, with an increasing P_2_O_5_ content, T_liq_ initially decreased and then increased, exhibiting a “V” trend. The lowest temperature was achieved in the C4P6 fraction, which is consistent with the experimentally measured FT trend. Figure 5b shows the Tend for different CaO/P_2_O_5_ ratios; a comparison reveals that C10P0 and C8P2 were dominated by anorthite, while C6P4 underwent a transition from anorthite to mullite. C4P6 experienced a transition from mullite to berlinite, and C2P8 and C0P10 were primarily dominated by berlinite. The high CaO content in C10P0 and C8P2 resulted in a preferential combination of a significant amount of Ca^2+^ with SiO_2_ and Al_2_O_3_ to form anorthite. As the total S + A content increased, the proportion of CaO and P_2_O_5_ in the system gradually decreased, leading to a transformation of minerals from calcium-based to skeletal and phosphate minerals, which led to a significant increase in T_liq_. Consequently, the last mineral solid phase partition mainly involved anorthite, mullite, and berlinite, the trend of Tend was as follows: T_Berlinite_ > T_Mullite_ > T_Anorthite_. This reasonably explains why the FT increased with the rising P_2_O_5_ content in the CaO/P_2_O_5_ ratio.

**Figure 6:**
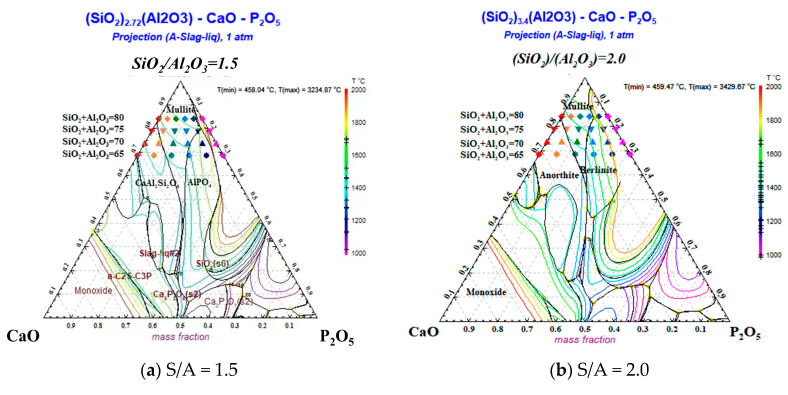

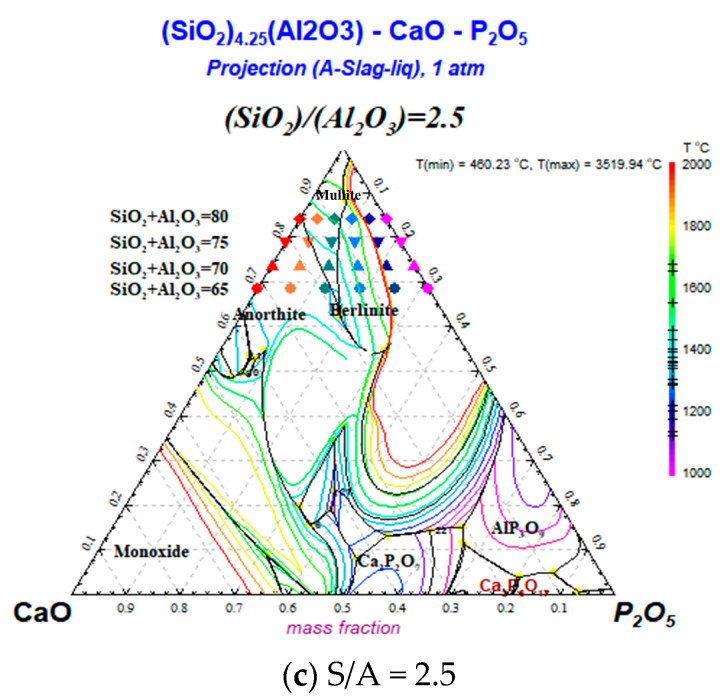
The quaternary phase diagrams of SiO_2_–Al_2_O_3_–CaO–P_2_O_5_ at three different Si/Al ratios. Each of the four rows of shapes in the ternary phase diagram corresponds to a different amount of S + A on the left, and the order of the shapes in each row from left to right is C10P0, C8P2, C6P4, C4P6, C2P8, and C0P10.

The thermodynamic properties of the slag and the direction of change can also be predicted by plotting the ternary phase diagram [26,27,28], as depicted in Figure 6. The simulation calculated the proposed ternary phase diagram phase region changes of 72 synthetic ashes in the SiO_2_–Al_2_O_3_–CaO–P_2_O_5_ quaternary system. Plotting the SiO_2_–Al_2_O_3_–CaO–P_2_O_5_ quadratic phase diagrams, the mass ratios of S/A (S/A = 1.5, 2.0, and 2.5) converted to molar ratios were 2.72, 3.4, and 4.25. At S/A = 1.5, with an increase in P_2_O_5_ content, the direction of the mineral phase was anorthite phase→mullite phase→berlinite phase→liquid phase; with an increase in the total amount of S + A, the phase was mainly concentrated in the mullite phase; with an increase in the S/A ratio and P_2_O_5_ content, the range of the phase was gradually narrowed and converged to the liquid phase. At S + A = 80, the mullite phase zone dominated. For SiO_2_+Al_2_O_3_ = 70 and S + A = 75, four phases were evenly distributed, and at S + A = 65, three phases dominated: the anorthite, berlinite, and liquid phases. The different-colored isotherm temperatures in Figure 6 correspond to the line temperatures of the liquid phase when the chemical reaction reached equilibrium, and it can be seen that the isotherm temperatures increased with the increase in the P_2_O_5_ content in the CaO/P_2_O_5_ ratio. Additionally, for the same CaO/P_2_O_5_ ratio, the isothermal temperature increased with the increasing S + A summation. This trend can also be reasonably explained by analyzing the FT trend from the perspective of the ternary phase diagram.

### 2.3. Construction and Verification of the Model

#### 2.3.1. Modeling the FT-I_a_ of Synthetic Ash

**Figure 7:**
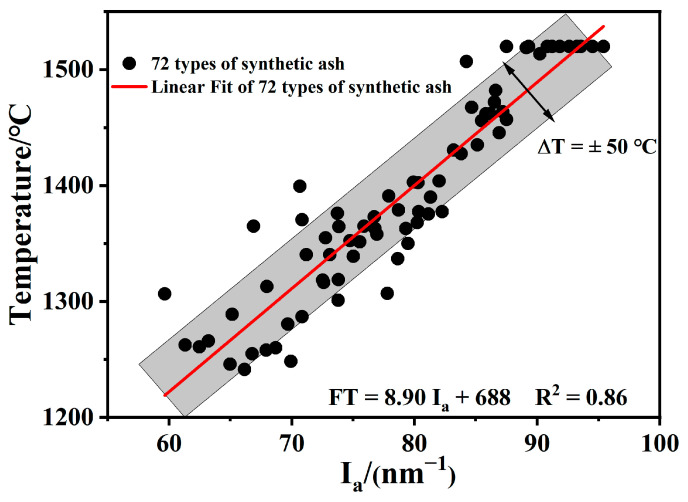
Correlation between measured FT and I_a_ of 72 synthetic ashes.

When constructing a model to judge whether a parameter is linearly correlated with FT, parameters such as the correlation coefficient (R, indicating the degree of correlation between the actual results and the predictions constructed by the model), the coefficient of determination (R^2^, the square of the correlation coefficient, a measure of the degree of fit of the model), and the range of deviation (ΔT, the degree of closeness of the putative predicted value of FTprediction with a different fit from the experimentally obtained FTmeasure) are generally used to reflect the degree of accuracy of the predictions [2,29]. The R-value corresponds to a range from −1 to 1; it is classified as a positive or negative correlation. For the prediction of the coal ash–slag system, |R| > 0.7 predicts the AFT, |R| > 0.8 indicates a higher correlation; and |R| > 0.9 suggests a high correlation between the parameters [30]. The closer R^2^ is to 1, the stronger the linear correlation between the two variables and the better the model is. When R^2^ is close to 0, there is no linear correlation between the two variables, and the model has poor predictive power [24]. The better they fit and the more accurate the predictions are, the smaller the value of ΔT and the smaller the difference between FT_prediction_ and FT_measure_ [31]. In this work, to respond to the predictive effect of the construction of the FT and I_a_ prediction models, two parameters, R^2^ and ΔT, were used. The I_a_ of 72 synthetic ashes and their FTs were combined by least-squares linear fitting to construct linear prediction formulae (Figure 7 and Equation (1)), all of which had R-values as high as 0.86 with a high correlation, and ΔT was generally within ±50 °C. The following universal prediction model was obtained:

FT = 8.90 I_a_ + 688   R^2^ = 0.86 (1)

#### 2.3.2. Validation 

To verify the feasibility of the FT-I_a_ prediction model, mixed ashes with different CaO/P_2_O_5_ ratios of co-gasification with phosphorus-rich biomass and coal in an S/A range of 1.5~2.5 and S + A = 65~80 were selected from the literature for prediction [7,8,9]. As reported in the literature, the ash compositions of 19 samples were determined by an X-ray fluorescence spectrometer with standard deviations of less than 0.05%. The ash components of mixed ashes were normalized and simplified into five types of oxides (SiO_2_, Al_2_O_3_, Fe_2_O_3_, CaO, and P_2_O_5_); the values of the parameters (S/A, S + A, CaO/P_2_O_5_, and I_a_), FT_measure_, FT_predication_, and ΔT are shown in Table 1.

**Figure 8:**
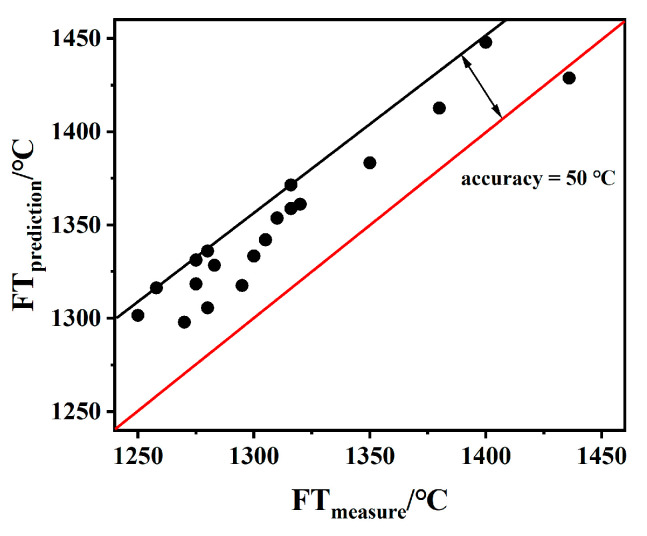
Comparison between FT_prediction_ and FT_measure_ from 10 literature data. The red line represents FT = FT; the black line represents the deviation of the predicted value from the experimental value data.

Figure 8 shows FT compared with the FTpredition for 19 ash samples. The comparison showed that the FT_predition_ was slightly higher than the FT_measure_, with the accuracy deviation in the range of ±40 °C, which suits the limitations of ASTM D 1857-04 (85 °C) [32], ISO 540-2008 (80 °C) [33], and GB/T219-2008 (80 °C) [34]. This means that the model is more suitable to predict phosphorus-rich biomass or mixed ash co-gasification with phosphorus-rich biomass and coal in an S/A range of 1.5~2.5 and S + A = 65~80.

## 3. Materials and Methods

### 3.1. Preparation of Synthetic Ashes

Coal ash is predominantly composed of silica and alumina, making it a silicate system with the inclusion of other fluxing components. The SiO_2_/Al_2_O_3_ ratio (S/A) is considered a crucial factor in determining the mineral transformation and melting temperature of coal ash [35]. The main components in coal ash are SiO_2_, Al_2_O_3_, CaO, and Fe_2_O_3_; the sum of the contents of these four oxides is greater than 90%. Phosphorus-rich biomass contains a large amount of CaO and P_2_O_5_; to eliminate the influence of other trace components, the experiment selected SiO_2_, Al_2_O_3_, Fe_2_O_3_, CaO, and P_2_O_5_ as the main components in the synthetic ash. Common coal/biomass ash is typically classified as low silica–alumina, medium silica–alumina, and high silica–alumina ash. Therefore, the total amounts of SiO_2_+Al_2_O_3_ are 65, 70, 75, and 80, and the S/A ratios are 1.5, 2.0, and 2.5, and 72 synthetic ashes were designed (as shown in Table 2). Fe_2_O_3_ was included at a concentration of 5%, which represents the average content in typical Chinese coal ash [36].

The work designed different CaO/P_2_O_5_ ratios (the mass ratios were 10:0, 8:2, 6:4, 4:6, 2:8, and 0:10, denoted as C10P0, C8P2, C6P4, C4P6, C2P8, and C0P10, respectively) on the total amount of the four different S + A constituents and different S/A ratios to investigate their effect on slag behavior. The five reagent-grade oxides mentioned above were placed into an agate mortar at the proportions specified in Table 2. Subsequently, they were thoroughly mixed and ground to ensure even distribution and homogeneity. The resulting mixture was heated in a muffle furnace at 815 °C, following the guidelines of the Chinese standard GB/T212-2008 [34]. After cooling, the mixture was returned to the agate mortar and ground thoroughly for 30 min until it reached a particle size of less than 75 μm.

### 3.2. Methods

#### 3.2.1. AFT Test

The AFTs of 72 synthetic ashes under a reducing atmosphere (CO/CO_2_, volume fraction: 3:2) were determined using an SDAF4000 auto-analyzer (Sundy, Changsha, China) according to the Chinese standard GB/T219-2008. The ash sample was made into a triangular cone and heated to 900 °C at a heating rate of 15 °C/min, and then to 1520 °C at 5 °C/min. The deformation temperature (DT), softening temperature, hemispherical temperature, and FT were recorded on the basis of the shape of the ash cone; at least three parallel experiments were performed for the AFTs of each sample, and the mean values were calculated and further analyzed and discussed.

#### 3.2.2. Method for Calculating the I_a_

The ionic potential can be expressed as the affinity energy of structure and charge in coal/biomass ash, the strength of bridge oxygen bond, which reflects the stability of coal ash structure. Five oxides of synthetic ash, SiO_2_, Al_2_O_3_, Fe_2_O_3_, CaO, and P_2_O_5_, were selected, and Equation (2) was used to calculate the I_a_ (as shown in Equation (2)) for the 72 synthetic ashes in Table 1 [16]:

(2) $$I_{a}=\frac{\sum {(x}_{i}\cdot I_{i})}{\sum x_{i}}$$

In the equation, x_i_ is the molar fraction of oxides normalized to the presence of Fe as Fe^2+^ under the high-temperature reducing atmosphere of the EFB gasifier [37], and Fe_2_O_3_ was converted to FeO for the calculation. I_i_ is the ionic potential of each metal ion, where I_Si_^4+^ = 95 nm^−1^, I_Al_^3+^ = 60.00 nm^−1^, I_Fe_^2+^ = 26.32 nm^−1^, I_Ca_^2+^ = 20.20 nm^−1^, and I_P_^5+^ = 147 nm^−1^.

I_a_ is a parameter that can accurately describe the chemical significance of the melt properties of coal (or biomass) ash or mixed ash. The magnitude of its value can represent the strength of polymerization of the silicate melt structure, which reflects the stability of the coal ash structure [18].

#### 3.2.3. Modeling of the FT-I_a_

Least-squares linear or fitting methods were used to fit the FT-I_a_ and construct the corresponding models. The least-squares linear fitting method is as follows [29]:

Assume that the linear equation             y = ax + b(3)

(4) $$\mathrm{Constructing}\;\mathrm{an}\;\mathrm{evaluation}\;\mathrm{function}\;L\left(a,b\right)=\sum_{i=1}^{N}\left[y_{i}-f(x_{i})\right]$$

(5) $$\mathrm{Determination}\;(R^{2})\;\;\;\;\;\;\;R^{2}=1-\frac{\sum_{i=1}^{n}{\left(y_{i}-{\hat{y}}_{i}\right)}^{2}}{\sum_{i=1}^{n}{\left(y_{i}-{\bar{y}}_{i}\right)}^{2}}$$

Deviation (ΔT)            ΔT = ±ΔT_max_ = ±∣FT_Predition_ − FT_measure_∣ (6)

where y is the FT; x is the I_a_; m is the sample capacity; x_i_, y_i_ are the x,y values for i group experiments; ${\bar{y}}_{i}$ is the mean value of y; ${\hat{y}}_{i}$ is the predicted value of y; FT_Predition_ is the predicted value of FT; FT_measure_ is the measured value of FT.

#### 3.2.4. FactSage Thermodynamic Simulation

FactSage thermodynamic software (version 7.3) was used to simulate the phase equilibria and mineralogical changes during the heating process of 72 synthetic ashes under a reducing atmosphere (CO/CO_2_ volume ratio = 3:2). In the “Equilib” module of the “FToxid” and “FactPS” databases, the evolution of minerals and their thermodynamic parameters (T_ini_, T_liq_, and T_end_) were calculated according to the principles of the Gibbs free energy minimization theory with the input of SiO_2_, Al_2_O_3_, Fe_2_O_3_, CaO, and P_2_O_5_; a selection of temperature ranges between 900 and 1800 °C; and a pressure of 1 atm. The synthetic ash was also simulated, and the change in the position of the phase region in the SiO_2_–Al_2_O_3_–CaO–P_2_O_5_ quadratic phase diagrams was calculated.

## 4. Conclusions

In this study, 72 synthetic ashes were prepared to reveal the influence of different CaO/P_2_O_5_ ratios on the FT of slag; an FT-I_a_ model relationship was constructed and used to predict the FT of 19 actual coal ashes, and the conclusions are as follows:

(1) With a decreasing CaO/P_2_O_5_ ratio, I_a_ gradually increased, and FT showed a stepwise upward trend; the mineral types in the ash changed in the direction of anorthite → mullite → berlinite, and the T_end_ temperature for the different types of minerals was T_Berlinite_ > T_Mullite_ > T_Anorthite_, resulting in an increased slag FT.
(2) When S + A was 65–80% and the S/A ratio is in the range of 1.5–2.5, FT and I_a_ are linearly correlated; the formula of this model is FT = 8.90 I_a_ + 688, which has a correlation coefficient of more than 0.86 and a deviation in the range of ±50 °C. Using the model proposed in this work, 19 samples were predicted within a deviation range of ±50 °C.
(3) The proposed correlation between FT and I_a_ can be used to predict the low-rank coal and phosphorus-rich biomass and their mixed ash, which provides a theoretical guide for the co-gasification of phosphorus-rich biomass and coal.

## Figures and Tables

**Table 1:** Ash compositions and parameters of 19 samples.

Samples	Ash Composition (wt/%)					S/A	S + A (wt/%) *^a^*	CaO/ P_2_O_5_	I_a_ (nm^−1^)	FT_measure_ (°C)	FT_predication_ (°C)	ΔT *^b^* (°C)
	SiO_2_	Al_2_O_3_	FeO	CaO	P_2_O_5_							
1	56.82	23.89	7.12	10.39	0.99	2.38	80.71	9.13:0.87	75.63	1320	1361.11	41.11
2	54.58	22.32	6.92	13.18	2.23	2.45	76.90	8.55:1.45	70.74	1295	1317.59	22.59
3	52.33	20.75	6.71	15.98	3.48	2.52	73.08	8.21:1.79	72.27	1275	1331.20	56.20
4	50.09	19.17	6.51	18.78	4.72	2.61	69.26	7.99:2.01	70.60	1258	1316.34	58.34
5	47.85	17.60	6.31	21.57	5.97	2.72	65.45	7.83:2.17	68.94	1250	1301.57	51.57
6	52.38	22.04	6.81	15.07	2.94	2.38	74.42	8.37:1.63	72.51	1300	1333.34	33.34
7	47.95	20.18	6.51	19.75	4.88	2.38	68.13	8.02:1.98	69.40	1280	1305.66	25.66
8	58.36	22.43	6.21	10.43	1.69	2.61	80.99	8.61:1.39	76.79	1316	1371.43	55.43
9	55.87	20.84	6.08	13.53	3.00	2.68	76.71	8.19:1.81	74.80	1310	1353.72	43.72
10	53.19	19.26	5.96	16.63	4.30	2.76	72.45	7.95:2.05	72.82	1280	1336.10	56.10
11	50.50	17.67	5.84	19.74	5.61	2.86	68.17	7.79:2.21	70.84	1275	1318.48	43.48
12	56.36	22.14	6.11	12.31	2.40	2.55	78.50	8.37:1.63	75.37	1316	1358.79	42.79
13	51.49	20.28	5.88	17.30	4.40	2.54	71.76	7.97:2.03	71.95	1283	1328.36	45.36
14	46.61	18.41	5.65	22.29	6.40	2.53	65.02	7.77:2.23	68.53	1270	1297.92	27.92
15	41.66	35.47	3.50	3.70	15.28	1.17	77.13	1.95:8.05	83.24	1436	1428.79	−7.21
16	39.21	33.38	3.30	3.48	20.26	1.17	72.59	1.47:8.53	85.38	1400	1447.91	47.91
17	45.12	28.68	15.57	4.59	4.31	1.57	73.8	5.16:4.84	73.49	1305	1342.06	37.06
18	46.92	33.73	4.54	7.15	7.16	1.39	80.65	5.00:5.00	78.12	1350	1383.27	33.27
19	43.18	29.02	8.50	13.92	4.43	1.49	72.2	7.59:2.41	81.42	1380	1412.68	32.68

*^a^* S + A = SiO_2_ + Al_2_O_3_; *^b^* ΔT = FT_predicaion_ − FT_meseare_.

**Table 2:** Ash compositions and parameters of 72 synthetic ashes.

S + A *^a^*	S/A	SiO_2_	Al_2_O_3_	Fe_2_O_3_	CaO/P_2_O_5_ Mass Ratio (wt. %)											
					C10P0 *^b^*		C8P2 *^c^*		C6P4		C4P6		C2P8		C0P10	
					CaO	P_2_O_5_	CaO	P_2_O_5_	CaO	P_2_O_5_	CaO	P_2_O_5_	CaO	P_2_O_5_	CaO	P_2_O_5_
65	1.5	39	26	5	30	0	24	6	18	12	12	18	6	24	0	30
	2.0	43.33	21.67													
	2.5	46.43	18.57													
70	1.5	42	28		25	0	20	5	15	10	10	15	5	20	0	25
	2.0	46.67	23.33													
	2.5	50	20													
75	1.5	45	30		20	0	16	4	12	8	8	12	4	16	0	20
	2.0	50	25													
	2.5	53.57	21.43													
80	1.5	48	32		15	0	12	3	9	6	6	9	3	12	0	15
	2.0	53.33	26.67													
	2.5	57.14	22.86													

*^a^* S + A = SiO_2_ + Al_2_O_3_; *^b^* C10P0: No P_2_O_5_ in this synthetic ash; *^c^* C8P2: CaO and P_2_O_5_ mass ratio 8:2.

## Data Availability

The data presented in this study are available upon request from the corresponding author.
